# The association between triglyceride-glucose index with gestational diabetes mellitus in pregnant women: a cohort study among Chinese women

**DOI:** 10.3389/fmed.2026.1541290

**Published:** 2026-03-31

**Authors:** Xin Zhao, Jianbin Sun, Ning Yuan, Xiaomei Zhang

**Affiliations:** Department of Endocrinology, Peking University International Hospital, Beijing, China

**Keywords:** gestational diabetes mellitus, glycosylated hemoglobin, oral glucose tolerance test, receiver operating characteristic, triglyceride-glucose index

## Abstract

**Background:**

This study aimed to investigate the association between the triglyceride and glucose (TyG) index and gestational diabetes mellitus (GDM) in pregnant women to prevent GDM.

**Methods:**

A total of 1,222 first-trimester pregnant women from the Obstetrics Department of Peking University International Hospital were enrolled in this prospective study between December 2017 and March 2019. The patients underwent an oral glucose tolerance test at 24–28 weeks of gestation and were regularly followed up until birth.

**Results:**

The TyG index was significantly higher in the GDM group than in the non-GDM group (*t* = −5.69, *P* < 0.05). In early pregnancy, the TyG index was positively associated with blood glucose levels at 0, 60, and 120 min before and after glucose loading (all *p* < 0.05). After adjusting for age, body mass index, parity, blood pressure, uric acid, and serum creatinine levels, the TyG index was found to be an independent risk factor for GDM. The model for predicting the risk of GDM using the TyG index showed an optimal cut-off point of 8.20.

**Conclusion:**

The TyG index serves as an independent risk factor for GDM and may predict the disease in pregnant women. Although the TyG index can be used as a marker of gestational diabetes, we should also pay more attention to fasting blood glucose and HbA1c levels. The TyG index may be integrated into early screening programs for GDM.

## Introduction

Gestational diabetes mellitus (GDM) is a common pregnancy-related complication that adversely affects the health of both mothers and infants (1). GDM exacerbates the risk of adverse perinatal outcomes in mothers and newborns, including preeclampsia, macrosomia, and neonatal hypoglycemia. Methods for the early detection and prediction of GDM are available, and exploring the risk factors associated with GDM development is of considerable clinical benefit (2). A previous study reported that the main pathogenesis of GDM is related to a decrease in insulin secretion and the development of insulin resistance (IR) during pregnancy (3, 4). The triglyceride–glucose (TyG) index, a marker of IR and GDM, has gained increasing attention.

The use of the TyG index to predict GDM risk has yielded inconsistent results. A recent meta-analysis included five cohort studies involving 382,213 female participants. The findings indicated that compared with women in the lowest TyG index group, those in the highest TyG index group had an independent positive correlation with the risk of GDM (OR = 2.5, 95% CI, 1.3, 4.7) (5). Interestingly, subgroup analysis revealed a significant correlation in Asian women (OR = 3.3, 95% CI 1.5, 7.3) but not in non-Asian women (OR = 1.0, 95% CI 0.4, 2.6). Thus, racial differences may exist in the association between the TyG index and GDM risk. A previous study has asserted that Asian women are more likely to suffer from GDM than Caucasians because the former seem to have limited insulin secretion compared to the latter (6). Similarly, an Australian study observed that after controlling for potential confounding factors, Chinese immigrant women were four times more likely to suffer from GDM than Australian-born Caucasian women (7). However, this meta-analysis includes only one study focusing on non-Asian women, and the Asian studies included do not provide specific cut-off values for the TyG index that can be used for clinical screening. Therefore, verifying the predictive efficacy of the TyG index in the Chinese population and establishing its diagnostic cut-off values are crucial for achieving early and low-cost screening of GDM. This study aims to explore the correlation between the TyG index in early pregnancy and the risk of GDM in Chinese pregnant women, determine its predictive efficacy and optimal cut-off values, and provide more operational evidence-based guidance for clinical prevention of GDM in Asian populations, especially Chinese women.

As GDM can be asymptomatic in its early stages, the TyG index—calculated from routinely measured first-trimester fasting glucose and triglycerides—can serve as a useful marker for predicting GDM risk, thereby facilitating early identification and intervention to prevent complications. This study aimed to explore the correlation between the TyG index in early pregnancy and the occurrence of GDM in pregnant Chinese women. Furthermore, this study aimed to identify the predictive factors for the occurrence of GDM and provide enhanced evidence for the clinical prevention of the disease.

## Materials and methods

The methods of our previous study were followed in this research (8).

### Research subject

This was a prospective study. A total of 1,222 pregnant women were enrolled in the Obstetrics Department of the Peking University International Hospital between December 2017 and March 2019. All pregnant women were enrolled at 7–12 weeks of gestation and were regularly followed up for fetal birth outcomes.

The inclusion criteria were as follows: (1) age > 18 years. (2) Will undergo checkups and deliveries at the hospital. (3) Acceptance of the relevant questionnaire survey and agreement to the collection of blood samples after being informed of the survey content.

The exclusion criteria were as follows: (1) unwillingness to undergo the oral glucose tolerance test (OGTT) at 24–28 weeks of gestation. (2) Diagnosis of cardiovascular, cerebrovascular, thyroid, hematological, liver, renal, or respiratory disease, or pre-pregnancy diabetes mellitus. (3) Multiple pregnancies. (4) Absence of basic data.

This study was approved by the Bioethics Committee of Peking University International Hospital. All protocols followed the ethical guidelines of the institution and national committee and complied with the 1964 Declaration of Helsinki and its subsequent amendments. All participants provided written informed consent. The ethics approval number was 2017-021 (BMR).

### Research methods

#### General information

Age, parity, and personal history of GDM were recorded at the time of enrolment in the first trimester. Blood pressure, including systolic blood pressure (SBP) and diastolic blood pressure (DBP), height, and weight were measured, and body mass index (BMI) was calculated and recorded. BMI was calculated using the following formula: BMI (kg/m^2^) = weight (kg)/body height^2^ (m^2^).

#### Biochemical index detection

All subjects fasted for 5 ml venous blood collected in the morning during 7–12 weeks of gestation in the first trimester. The detection indices included glycosylated hemoglobin (HbA1c), fasting blood glucose (FBG), total cholesterol (TC), triglycerides (TG), high-density lipoprotein cholesterol (HDL-C), low-density lipoprotein cholesterol (LDL-C), uric acid (UA), serum creatinine (SCr), homocysteine (Hcy), and lipoprotein a (LPA). The chemiluminescence method was used to test blood glucose and lipid profiles. HbA1c levels were measured using high-performance liquid chromatography (HPLC) with a Dongcao G8 analyzer.

The TyG index was calculated using the following formula: TyG = ln (TG × FBG/2). The third percentile of TyG was divided into three groups: low (<7.9), middle (7.9–8.3), and high (≥8.3).

#### Diagnosis of GDM

Pregnant women were screened for GDM using a 75 g OGTT at 24–28 weeks of gestation in the second trimester. Pregnant women were admitted to the hospital in the morning after fasting for 8–12 h. They were administered 75 g of glucose powder dissolved in 250–300 ml of warm boiled water orally within 5 min. Blood glucose levels before glucose water intake (GLU_0min_), 1 h (GLU_60min_) after glucose water intake, and 2 h (GLU_120min_) after glucose water intake were measured.

The International Association of Diabetes and Pregnancy Study Groups (IADPSG) criteria were used to diagnose GDM (9), that is, the GLU_0min_, GLU_60mi*n*_, and GLU_120min_ values should be lower than 5.1, 10.0, and 8.5 mmol/L, respectively. GDM was diagnosed if any blood glucose value exceeded the criteria. We excluded patients with overt diabetes mellitus during pregnancy.

Gestational weight at 24–28 weeks was recorded, and weight gain was calculated and recorded.

### Statistical analysis

All data were analyzed using SPSS version 22.0. Data were tested for normality, and those that were normally distributed were expressed as means ± standard deviation (x ± s) and compared using *t*-tests. The counting data were expressed as rates and compared between the two groups using the χ2 test. Furthermore, Pearson’s correlation coefficient was used to determine the association between the biochemical indices and blood glucose levels before and after glucose loading. Unconditional logistic regression models were employed to perform a univariate analysis of independent variables for GDM to calculate the OR and 95% CI. To analyze the correlation between TyG and GDM, multivariate logistic regression analysis was performed using GDM as the dependent variable and variables that were significant in the univariate analysis as independent variables. The adjusted variables included those that were statistically significant in the univariate regression and those that were closely related to the occurrence of GDM in clinical practice. Receiver operating characteristic (ROC) curves were plotted, and the area under the curve (AUC) was calculated for each model.

The AUC were compared using DeLong’s test. The maximum value of the Youden index corresponded to the optimal diagnostic threshold of the method, which was the cut-off. All statistical tests were two-sided, and statistical significance was set at *P* < 0.05.

## Results

### Comparison of general conditions and biochemical indexes between the two groups in the first pregnancy and oral glucose tolerance test results

Of the 1,222 patients, 231 were diagnosed with GDM during the second trimester, with an incidence of 18.9%. Women in the GDM group had a higher body mass index (BMI) than those in the non-GDM group and also exhibited higher levels of hemoglobin A1c and fasting blood glucose (FBG) in the first trimester of pregnancy (all *p* < 0.05). The TyG index was significantly higher in the GDM group than in the non-GDM group (*t* = −5.69, *p* < 0.05). In addition, the proportion of women with a personal history of GDM was significantly higher in the GDM group than in the non-GDM group (χ2 = 10.3, *p* < 0.05). Moreover, the proportion of multiparous women was significantly higher in the GDM group than in the non-GDM group (χ2 = 9.9, *p* < 0.05). The levels of triglycerides (TG), total cholesterol (TC), low-density lipoprotein cholesterol (LDL-C), and uric acid (UA) were also higher in the GDM group than in the non-GDM group (all *p* < 0.05) (Table 1).

**TABLE 1:** Comparison of general conditions and biochemical indexes between the two groups in the first trimester pregnancy and oral glucose tolerance test (OGTT) results.

Index	Non-GDM group (*n* = 991)	GDM group (*n* = 231)	t(X^2^)	*P*
Age (years)	30.95 ± 3.75	30.77 ± 3.43	0.65	0.51
BMI (kg/m^2^)	21.84 ± 2.94	22.47 ± 3.37	−7.65	<0.01
Personal history of GDM	10 (1.09%)	43 (18.61%)	10.32	<0.01
Parity				
0	584 (58.93%)	111 (48.05%)	9.93	<0.01
≥1	407 (41.07%)	120 (51.95%)		
SBP (mmHg)	110.03 ± 10.60	109.82 ± 10.58	0.47	0.65
DBP (mmHg)	66.21 ± 8.94	65.96 ± 8.94	2.63	<0.01
TC (mmol/L)	3.94 ± 0.69	4.11 ± 0.67	−2.72	<0.01
TG (mmol/L)	0.96 ± 0.51	1.10 ± 0.73	−4.46	<0.01
LDL-C (mmol/L)	2.05 ± 0.55	2.05 ± 0.51	−2.32	0.02
HDL-C (mmol/L)	1.42 ± 0.29	1.42 ± 0.25	−0.51	0.61
UA (μmol/L)	213.55 ± 45.57	220.61 ± 51.51	−4.69	<0.01
sCr (μmol/L)	49.57 ± 6.99	49.43 ± 7.40	1.33	0.18
HbA1c (%)	5.21 ± 0.20	5.32 ± 0.31	−5.40	<0.01
FBG (mmol/L)	4.87 ± 0.40	5.04 ± 0.41	−6.18	<0.01
HCY (μmol/L)	6.63 ± 3.41	6.50 ± 2.60	1.12	0.26
LPA (mg/L)	152.32 ± 45.43	148.07 ± 54.32	0.31	0.76
Gestational weight gain (kg)	9.23 ± 1.19	12.0 ± 2.25	−5.03	<0.01
TyG	8.11 ± 0.38	8.30 ± 0.48	−5.69	<0.01
GLU_0min_	4.50 ± 0.32	4.96 ± 0.57	−17.52	<0.01
GLU_60min_	7.31 ± 1.30	9.71 ± 1.57	−23.79	<0.01
GLU_120min_	6.60 ± 1.01	8.47 ± 1.59	−22.54	<0.01

BMI, body mass index; SBP, systolic blood pressure; DBP, diastolic blood pressure; FBG, fasting blood glucose, HbA1c, glycosylated hemoglobin; sCr, serum creatinine; UA, uric acid; TC, total cholesterol; TG, triglycerides; LDL-C, low-density lipoprotein cholesterol; HDL-C, high-density lipoprotein cholesterol; GLU_0min_, fasting blood glucose before OGTT; GLU_60min_, blood glucose 60 min after OGTT; GLU_120min_, blood glucose 120 min after OGTT; TyG, triglyceride–glucose; Hcy, homocysteine; LPA, lipoprotein a.

### Association between the TyG and blood glucose level before and after glucose loading

**FIGURE 1:**
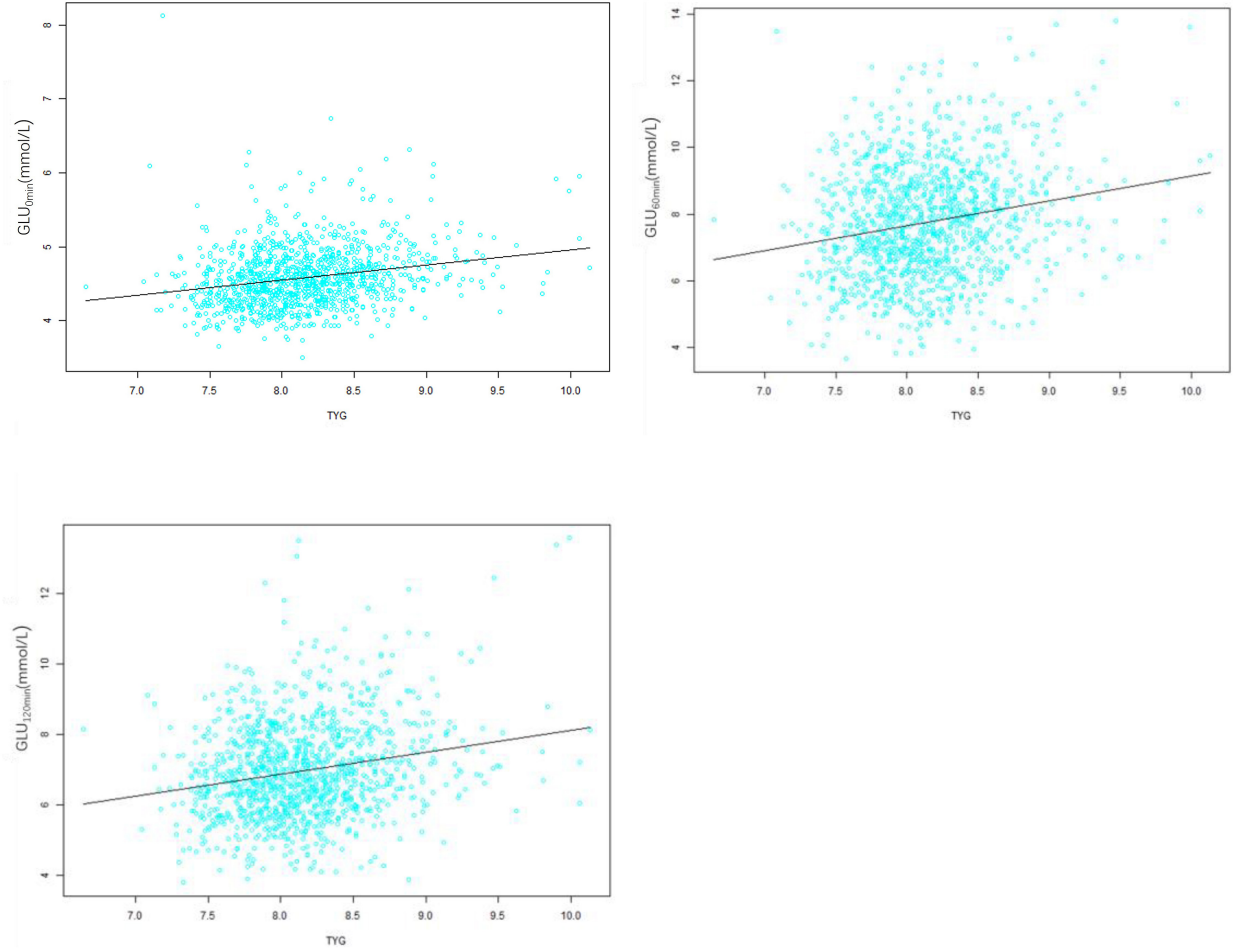
Correlation analysis between the triglyceride-glucose (TyG) index and blood glucose levels at 0, 60, and 120 min during the oral glucose tolerance test (OGTT). The positive correlations were evident between the TyG index and blood glucose levels before and after glucose loading (*r* = 0.23, *r* = 0.20, *r* = 0.21, respectively).

Positive correlations were evident between the TyG index and blood glucose levels before and after glucose loading (all *p* < 0.05) (Figure 1). This indicates that with an increase in TyG levels, blood glucose levels before and after the glucose load also increase.

### Logistic regression analysis of TyG and GDM

Multivariate logistic regression analysis was performed using GDM as the dependent variable and variables that were significant in the univariate analysis as independent variables. After adjusting for age, BMI, parity, blood pressure, and UA levels, the TyG index was found to be an independent risk factor for GDM (Table 2). We conducted a collinearity test on the independent variable, and the VIF value of the independent variable was 4.8.

**TABLE 2:** Logistic regression between TyG in first trimester with GDM.

Index	Unadjusted OR	95% CI	*P*	Adjusted OR	95% CI	*P*
Age (years)	0.89	0.88, 1.00	0.51	0.91	0.87, 1.01	0.59
BMI (kg/m^2^)	1.21	1.03, 1.24	<0.01	1.18	1.08, 1.28	0.03
SBP (mmHg)	0.97	0.86, 1.03	0.68	1.05	0.96, 1.04	0.41
UA (μmol/L)	0.95	0.92, 1.03	0.01	1.04	1.01, 1.10	0.02
sCr (μmol/L)	1.03	0.87, 1.13	0.23	1.03	0.92, 1.04	0.10
Gestational weight gain (kg)	1.27	1.03, 1.68	0.01	1.23	1.02, 1.56	0.02
HCY (μmol/L)	0.91	0.81, 1.02	0.29	0.91	0.82, 1.00	0.09
LPA (mg/L)	1.03	1.01, 1.06	0.65	1.07	1.03, 1.13	0.47
Parity						
≥1	1.57	1.17,2.07	<0.01	1.58	1.18, 2.03	<0.01
TyG	2.27	1.70,3.06	<0.01	1.81	1.28, 2.53	<0.01
Low level (<7.9)	1			1		
Middle level (7.9–8.3)	1.11	0.75, 1.63	0.61	0.91	0.60, 1.37	0.64
Higher level (>8.3)	2.11	1.49, 3.03	<0.01	1.55	1.07, 2.28	0.01

BMI, body mass index; SBP, systolic blood pressure; sCr, serum creatinine; UA, uric acid; TyG, triglyceride–glucose; Hcy, homocysteine; LPA, lipoprotein a.

### Multivariate prediction model for GDM

**FIGURE 2:**
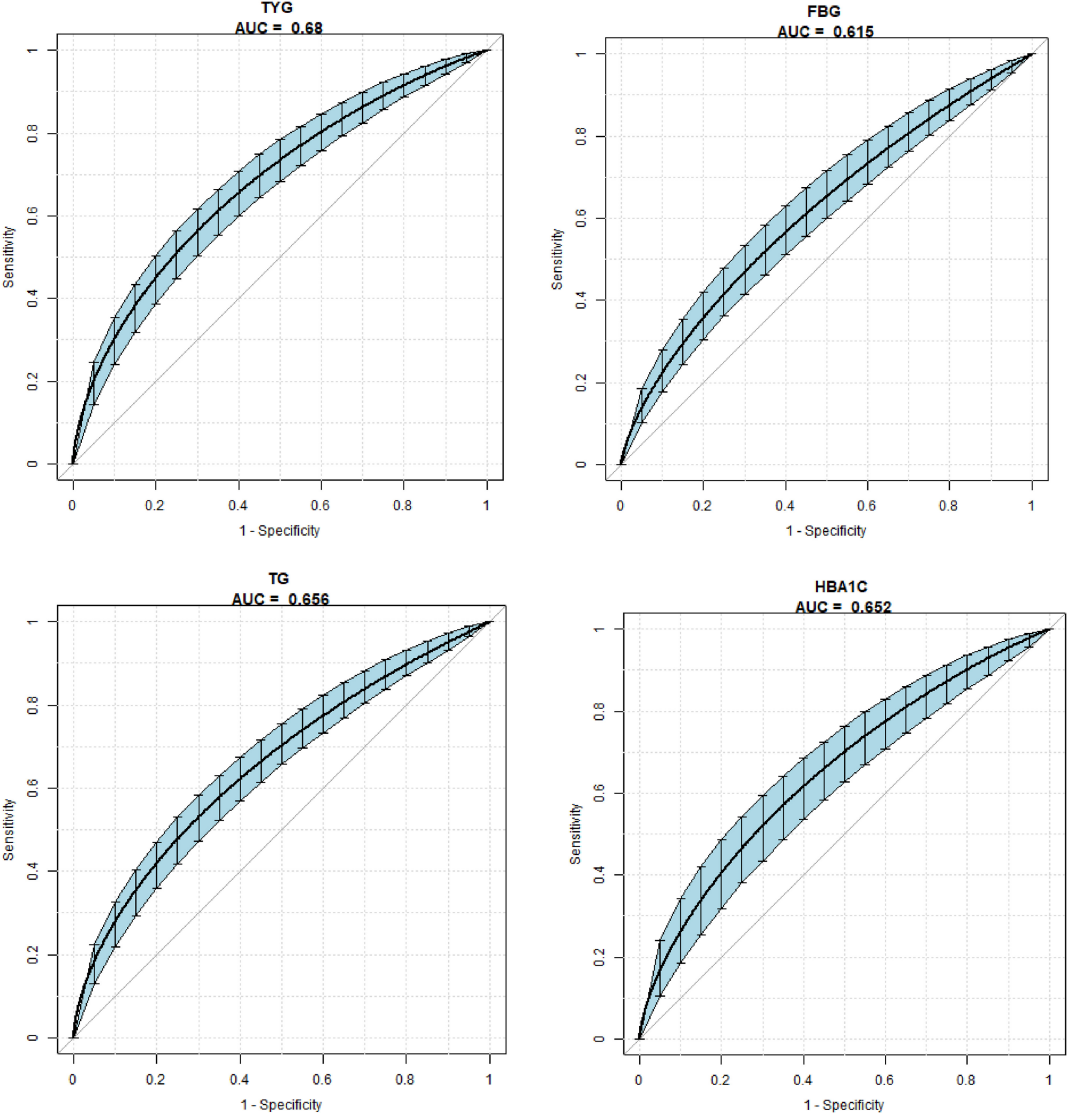
The overall predictive accuracy of triglyceride-glucose (TyG) was 0.68 (95% CI 0.64, 0.71). The overall predictive accuracy of fasting blood glucose (FBG) was 0.62 (95% CI 0.57, 0.66). The overall predictive accuracy of TG 0.66 (95% CI 0.62, 0.69). The overall predictive accuracy of HbA1c 0.65 (95% CI 0.60, 0.70).

The overall predictive accuracy of TyG was 0.68 (95% CI 0.64, 0.71). The overall predictive accuracy of FBG was 0.62 (95% CI 0.57, 0.66). The overall predictive accuracy of TG 0.66 (95% CI 0.62, 0.69). The overall predictive accuracy of HbA1c 0.65 (95% CI 0.60, 0.70). The AUCs of the models used to predict the risk of GDM using the four variables of TG, FBG, and TyG were TyG > TG > HbA1c > FBG. The “cut-off” is the value corresponding to the highest diagnostic accuracy of a variable for GDM. The model to predict the risk of GDM using TyG showed that the cut-off value of the TyG index for predicting GDM was 8.20 (Figure 2 and Table 3).

**TABLE 3:** Univariate predictive models of gestational diabetes mellitus (GDM) with TyG.

Index	AUC (95% CI)	Specificity (95% CI)	Sensitivity (95% CI)	Cut-off
TyG	0.68 (0.64, 0.71)	0.63 (0.58, 0.74)	0.61 (0.59, 0.72)	8.20
FBG	0.62 (0.57, 0.66)	0.71 (0.70, 0.72)	0.50 (0.41, 0.61)	5.05
TG	0.66 (0.62, 0.69)	0.75 (0.70, 0.81)	0.50 (0.46, 0.67)	1.09
HbA1c	0.65 (0.60, 0.70)	0.81 (0.74, 0.88)	0.42 (0.40, 0.56)	5.42

TyG, triglyceride-glucose; FBG, fasting blood glucose; TG, triglyceride.

**FIGURE 3:**
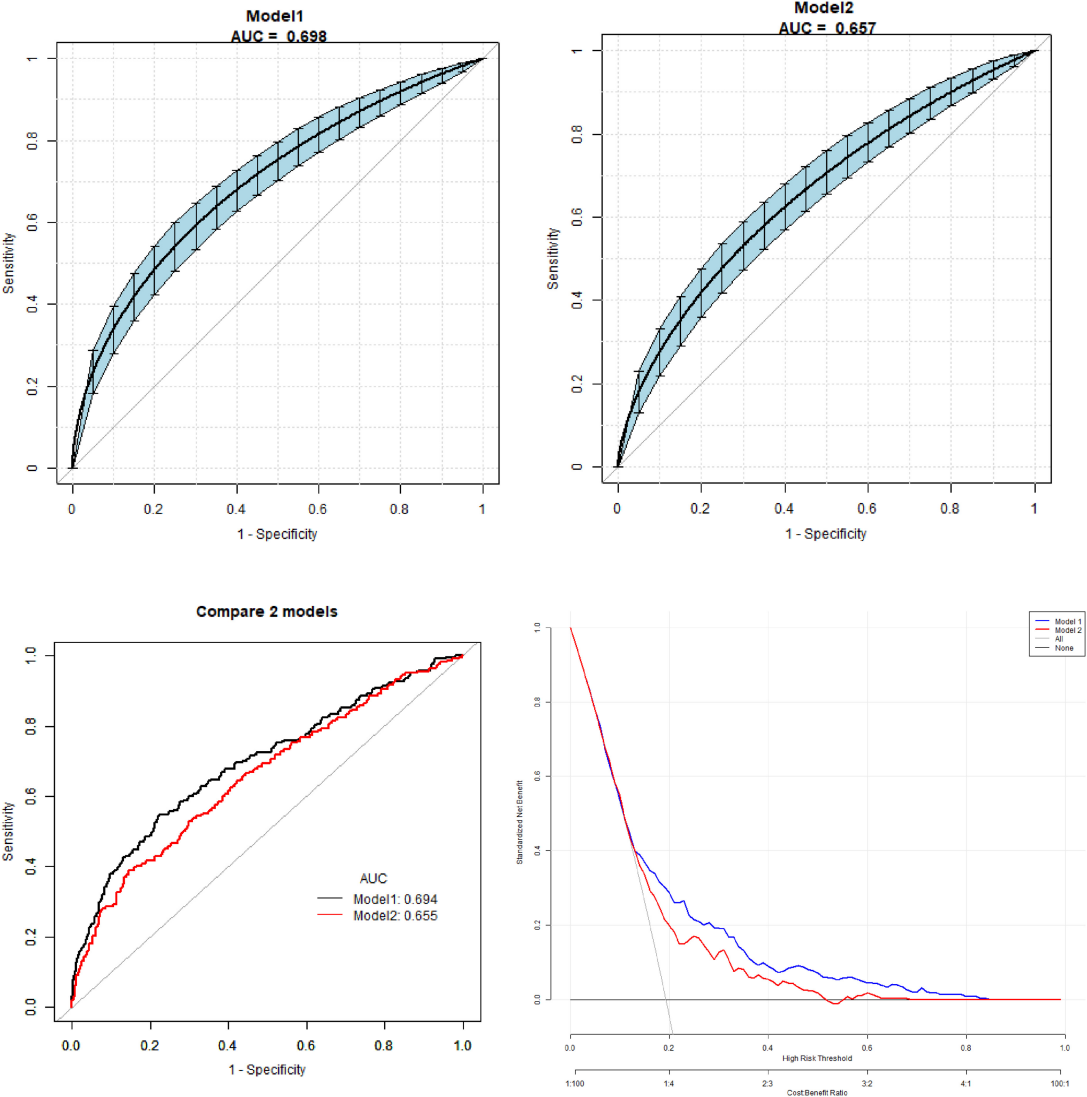
The area under the curve (AUC) of model 1 for predicting gestational diabetes mellitus (GDM) including triglyceride-glucose (TyG) is 0.70 (95% CI 0.64, 0.73). The specificity of receiver operating characteristic (ROC) is 0.81 (95% CI 0.74, 0.88), a sensitivity of 0.61 (95% CI 0.48, 0.66) and an accuracy of 0.69 (95% CI 0.68, 0.82). The AUC of model 2 for predicting GDM without TyG is 0.66 (95% CI 0.60, 0.71). The specificity of ROC is 0.85 (95% CI 0.79, 0.89), a sensitivity of 0.41 (95% CI 0.38, 0.52) and an accuracy of 0.66 (95% CI 0.61, 0.69). DeLong’s test results suggested that there was statistically significant difference in the predictive performance between the two models for GDM (*p* < 0.05).

In the multivariate predictive model, Model 1 was established with GDM as the dependent variable and age, BMI, parity, blood pressure, UA level, and TyG index as the independent variables. The AUC of model 1 for predicting GDM including TyG is 0.70 (95% CI 0.64, 0.73). The specificity of ROC is 0.81 (95% CI 0.74,0.88), a sensitivity of 0.61 (95% CI 0.48,0.66) and an accuracy of 0.69 (95% CI 0.68,0.82). Model 2 was established with GDM as the dependent variable and age, BMI, parity, blood pressure, and UA as independent variables. The AUC of model 2 for predicting GDM without TyG is 0.66 (95% CI 0.60, 0.71). The specificity of ROC is 0.85 (95% CI 0.79, 0.89), a sensitivity of 0.41 (95% CI 0.38, 0.52) and an accuracy of 0.66 (95% CI 0.61,0.69). The sensitivity and specificity of GDM prediction increased significantly after adding the TyG index to the model, and the AUC with the TyG index was significantly higher than that without it. DeLong’s test results suggested a statistically significant difference in the predictive performance between the two models for GDM (*p* < 0.05). Further pairwise DeLong’s tests revealed that the AUC of the TyG index was significantly higher than that of FBG (*p* < 0.01) and HbA1c (*p* = 0.03), but the difference compared to TG did not reach statistical significance (*p* = 0.08) (Figure 3).

## Discussion

Gestational diabetes mellitus is a common complication of pregnancy, affecting approximately 1/6 of pregnant women worldwide (1). Although blood glucose levels in patients with GDM usually return to normal after delivery, those previously diagnosed with GDM have an increased risk of developing T2DM in subsequent pregnancies (2). A recent Indian study reported that IR measured using the homeostatic model assessment of insulin resistance (HOMA-IR) is an independent predictor of postpartum dysglycemia in women with GDM (10, 11). Therefore, prompt diagnosis of IR in pregnant women is crucial, and interventions to alleviate it should be initiated early in pregnancy and continue postpartum.

In recent years, the association between the TyG index and the increased risk of cardiovascular diseases and T2DM in low- and medium-income countries has been explained by the increased vulnerability of these populations to IR (12). During pregnancy, owing to the particularity of women, performing the hyperinsulinemic–euglycemic clamp (HIEC) test to determine whether IR is combined is impossible; however, the TyG index is a reliable and convenient alternative indicator of IR, which is derived from FBG and TG (13). Compared with the gold standard HIEC, TyG index as an IR index has high sensitivity (96.5%) and specificity (85.0%) (14), and compared with HOMA-IR, it has good sensitivity. Several studies in the United States, Europe, and Asia have shown that although ectopic fat deposition is affected by race, the TyG index is significantly correlated with IR (15).

The findings of this study demonstrated that the TyG index was significantly higher in the GDM group than in the non-GDM group (*t* = −5.69, *p* < 0.05). After adjusting for age, BMI, parity, blood pressure, UA, and sCr, this index was found to be an independent risk factor for GDM. Pazhohan et al. (16) examined the relationship between the maternal first-trimester plasma lipid profile, FBG, and TyG index and the risk of GDM. After adjusting for potential confounders, the relative risk of GDM in women in the top tertile of the TyG index was 4.9-fold higher than that in women in the bottom tertile. Similarly, another study established that the mean TyG index value in the GDM group was significantly higher than that in the non-GDM group (4.9 ± 0.7 vs. 4.7 ± 0.2, *p* < 0.001). A sensitivity of 89% (95% CI: 0.8, 1.0) and a specificity of 50% (95% CI: 0.4, 0.6) were observed, accompanied by a high negative predictive value of 93% (17). The sample size was small in previous studies, and the sample size of our study was the largest among all studies. Another study (18) with a large sample size reported that an increased pre-registration TyG index was associated with the risk of GDM and concluded that this index may be an early marker of the disease. However, the duration for which the TyG index was tested before pregnancy was not specified in the study. FBG and TG levels were affected by diet and exercise status in the weeks prior to detection. Moreover, the TyG index measured before pregnancy did not represent the index during gestation. Although the results indicated that the index could be used as a predictor of GDM, the study was significantly biased. The subjects of this study were pregnant women. Using the TyG index in the first trimester of pregnancy as a predictor of GDM in the second trimester can more accurately reflect the association between IR and GDM during pregnancy than using BMI.

Recent investigations have alluded that in different ethnic groups, such as South Koreans, Singaporeans, and Europeans (19, 20), the TyG index is positively correlated with the risk of T2DM. In addition, the relationship between the TyG index and T2DM risk was non-linear, with the slope of the curve increasing with an increase in the TyG index (21). Furthermore, in individuals with normal FBG levels, the TyG index value for predicting T2DM risk has been perceived to be better than that of TG or FBG (20, 22). Compared with other IR indicators, such as the TG/high-density lipoprotein cholesterol ratio and HOMA-IR, the TyG index has been confirmed to be a better tool for predicting the development of DM (23).

A meta-analysis of five studies revealed that a high TyG index may independently predict the risk of GDM in Asian women (5). However, the two Asian studies did not reveal the cut-off point of the TyG index to predict the risk of GDM. Using single-factor regression analysis, we established a prediction model for GDM, which included the TyG index, and observed that the cut-off value of the index for predicting the risk of GDM was 8.20. This is the first study to report TyG as a cut-off point for predicting GDM in pregnant Chinese women.

To better use early pregnancy glucose and lipid metabolism indicators in the prediction of GDM risk, a logistic regression model was applied to establish a predictive model. In our study, after adding the TyG index to the model, the sensitivity and specificity of GDM prediction were significantly enhanced, and the AUC with the TyG index was significantly higher than that without it. A variance inflation factor of 4.8 indicated moderate multicollinearity among the independent variables. To assess its potential impact on model stability, we examined the standard errors of the coefficient estimates, which were within acceptable ranges. Furthermore, the core finding that TyG is an independent risk factor for GDM remained robust when any single variable was removed from the model. Therefore, we concluded that the logistic regression model provided stable estimates for our primary conclusion.

This longitudinal study involved a sample size of 1,222 pregnant women, which was much higher than that of previous studies. The results of this study demonstrated that the overall predictive accuracy of the TyG index was 0.70 (95% CI: 0.64, 0.73), suggesting that this index determined in early pregnancy has a certain predictive value for GDM. Although the AUC index was only 0.70, it still showed that the TyG index in the first trimester of pregnancy had predictive value for GDM. In our future research, we plan to further expand the sample size, which may potentially lead to an increase in the AUC. The findings of this study further confirmed that the TyG index in the first trimester was an independent risk factor for GDM in the second trimester. This study further clarified the cut-off value of the TyG index for predicting GDM, which holds clinical significance for predicting the risk of GDM in pregnant women. In addition, being a non-insulin-based index, it is less expensive than insulin-based markers. FBG and TG levels are typically used as routine indicators during early pregnancy. At the public health level, the TyG index can be used as a measure of IR in early pregnancy without increasing costs. Therefore, this index is an attractive alternative to the IR and can be widely used in pregnant women. In this study, adding the TyG index to a model containing traditional risk factors increased the AUC from 0.66 to 0.70. Although this absolute improvement is modest, it is statistically significant. More importantly, from a clinical perspective, this enhancement was driven by a marked increase in model sensitivity (from 0.41 to 0.61), which is highly valuable for an early screening strategy aimed at minimizing missed diagnoses in a public health context.

Meanwhile, in the regression analysis, our results showed that HbA1c and FBG levels had a higher correlation with GDM. Therefore, we believe that TyG index is a predictive indicator of GDM in early pregnancy; however, we still need to focus on HbA1c and FBG levels in early pregnancy. Furthermore, in the multivariate regression model, HbA1c and FBG levels maintained strong independent associations with GDM (Table 2), reaffirming the central role of glycemic measures in GDM diagnosis. Our findings suggest that in addition to focusing on traditional glycemic indicators (HbA1c and FBG), incorporating the TyG index—a marker of insulin resistance—can provide additional, independent predictive information for GDM risk. This is particularly relevant for identifying pregnant women who may have normal fasting glucose but underlying metabolic risk in early pregnancy.

The stronger predictive performance of the TyG index in our Chinese cohort aligns with the hypothesis that Asian populations may be more susceptible to GDM due to a predisposition toward lower insulin secretory capacity rather than severe insulin resistance. In this context, the TyG index, as a surrogate for insulin resistance, might capture an early, compensatory metabolic shift that precedes significant beta-cell dysfunction. Our finding of a significant association even after adjusting for key confounders supports the idea that assessing insulin resistance early in pregnancy could be particularly informative for GDM risk stratification in populations with this phenotypic trait.

This study suggests that the first-trimester TyG index, with a cut-off of 8.20, could serve as an independent predictor for GDM in Chinese women. From a clinical implementation perspective, the TyG index holds significant promise due to its practicality and cost-effectiveness. It can be seamlessly integrated into existing prenatal care pathways by simply calculating it from fasting glucose and triglyceride levels, which are routinely measured in first-trimester blood work, requiring no additional blood draws or costs. Before widespread adoption, however, the external validity of this cut-off value must be confirmed in larger, multi-center cohorts across diverse regions of China. Future research should focus on: (1) validating the proposed cut-off in independent, multi-center populations; (2) investigating the dynamic trajectory of the TyG index throughout pregnancy and its relationship with GDM progression; and (3) developing and validating integrated prediction models that combine the TyG index with other clinical parameters to further improve risk stratification and guide personalized interventions.

This study had several limitations. First, this study was a single-center design. Although the sample size (*n* = 1,222) is relatively larger than that of most previous similar studies, the generalizability of our findings to the broader national population may still be limited. Hence, in future research, we will include 20 research hospital centers in China to further expand the sample size and explore the predictive role of TyG in GDM. Second, only the TyG index in the first trimester was included, and it was not dynamically evaluated throughout the entire pregnancy. Third, this study lacks external validation. To address this and facilitate the clinical translation of the TyG index, we have planned a subsequent multi-center validation study involving five hospitals from northern, southern, and eastern China. This study aims to: (1) externally validate the diagnostic performance and optimal cut-off value (8.20) of the first-trimester TyG index established in the current cohort; and (2) develop and validate a comprehensive GDM risk prediction nomogram incorporating the TyG index, basic clinical characteristics, and gestational weight gain. Finally, this study did not use external validation methods to test the sensitivity and specificity of TyG index. Hence, in future investigations, we plan to include the TyG index at different pregnancy stages in an external population to clarify its association with GDM.

## Conclusion

Analysis of the correlation between the TyG index and GDM in pregnant women revealed that the index in the first trimester was an independent risk factor for GDM. In the regression analysis, our results showed that HbA1c and FBG levels were more strongly correlated with GDM than other factors. Although the TyG index can be used as a marker of gestational diabetes, we should also pay more attention to FBG and HbA1c levels.

## Data Availability

The data analyzed in this study is subject to the following licenses/restrictions: data used to support the findings of this study are available from the corresponding author upon request. Requests to access these datasets should be directed to zhaoxin2012@aliyun.com.
